# Heme Oxygenase-1 Supports Mitochondrial Energy Production and Electron Transport Chain Activity in Cultured Lung Epithelial Cells

**DOI:** 10.3390/ijms21186941

**Published:** 2020-09-22

**Authors:** Jennifer F. Carr, David Garcia, Alejandro Scaffa, Abigail L. Peterson, Andrew J. Ghio, Phyllis A. Dennery

**Affiliations:** 1 Department of Molecular Biology, Cell Biology and Biochemistry, Brown University, Providence, RI 02906, USA; jennifer_carr@brown.edu (J.F.C.); abigail_peterson@brown.edu (A.L.P.); 2 Department of Chemistry, Brown University, Providence, RI 02906, USA; david_garcia@alumni.brown.edu; 3 Department of Molecular Pharmacology, Physiology, and Biotechnology, Brown University, Providence, RI 02906, USA; alejandro_scaffa@alumni.brown.edu; 4 National Health and Environmental Effects Research Laboratory, US Environmental Protection Agency, Chapel Hill, NC 27599, USA; Ghio.Andy@epa.gov; 5 Department of Pediatrics, Warren Alpert Medical School, Brown University, Providence, RI 02903, USA; 6 Hasbro Children’s Hospital, Providence, RI 02903, USA

**Keywords:** metabolism, succinate dehydrogenase, iron, heme

## Abstract

Heme oxygenase-1 is induced by many cellular stressors and catalyzes the breakdown of heme to generate carbon monoxide and bilirubin, which confer cytoprotection. The role of HO-1 likely extends beyond the simple production of antioxidants, for example HO-1 activity has also been implicated in metabolism, but this function remains unclear. Here we used an HO-1 knockout lung cell line to further define the contribution of HO-1 to cellular metabolism. We found that knockout cells exhibit reduced growth and mitochondrial respiration, measured by oxygen consumption rate. Specifically, we found that HO-1 contributed to electron transport chain activity and utilization of certain mitochondrial fuels. Loss of HO-1 had no effect on intracellular non-heme iron concentration or on proteins whose levels and activities depend on available iron. We show that HO-1 supports essential functions of mitochondria, which highlights the protective effects of HO-1 in diverse pathologies and tissue types. Our results suggest that regulation of heme may be an equally significant role of HO-1.

## 1. Introduction

The clinical administration of supplemental oxygen to premature babies is sometimes necessary to support life as their lungs and alveoli are not completely developed. Administration of oxygen at concentrations higher than ambient (>21%) has its own negative consequences owing to the toxic effects of reactive oxygen on tissues, cells, and their subcellular building blocks. Upon oxidative damage or injury the pro-oxidant heme molecule may be released, causing further damage [1]. One defense mechanism in the lung is induction of anti-oxidant programs including increased expression and activity of heme oxygenase-1 (*hmox1*, HO-1) [2,3]. HO-1 is the first and rate-limiting enzyme involved in the breakdown of heme. The end products of heme catalysis include the antioxidant molecule bilirubin, the anti-inflammatory CO, and ferrous iron (Fe^2+^), which is sequestered by ferritin. Studies have demonstrated the protective effects of HO-1 at the organismal level, for example in acute lung injury, as well as the cellular level, by reducing damage caused by reactive oxygen species (ROS) [4,5]. The importance of HO-1 is highlighted by a mouse model of HO-1 deficiency, characterized by significant embryonic lethality, in which survivors have increased markers of inflammation, have low serum iron, and increased iron deposition in the liver and kidneys [6].

Despite comprehensive investigation, the mechanism of HO-1 protection is still not well understood. Under normal, non-hemolytic conditions, the amount of cellular labile heme in animals is in the low micromolar range in red blood cells and even lower in non-erythroid cells [7,8]. Therefore, the role of HO-1 is likely more complex and may extend beyond the products of heme catalysis. In particular HO-1, originally classified as a heat shock protein, is upregulated under myriad stresses including exposure to heavy metals, hyperoxia, and hypoxia, in the absence of elevated heme [9]. The broad spectrum of inducers hints that there may be multiple and varied targets of action. In one study, at baseline, cells overexpressing HO-1 did not show increased ferritin expression, but did so when challenged with hemin, suggesting the amount of heme limits injury, not HO-1 [10]. Additionally catalytically inactive forms of HO-1 have been shown to confer cytoprotection [11,12,13].

HO-1 activity has also been tied to metabolism in several ways. In mice, induction of HO-1 resulted in improved insulin sensitivity and adipose tissue remodeling [14,15]. In diabetic rats mitochondrial transporters such as citrate and ADP/ATP carriers are reduced compared to non-diabetic controls, but levels of these transporters were increased upon induction of HO-1 [16]. Biosynthesis of heme is complex and starts and ends in the mitochondria where heme is an essential cofactor in the mitochondrially encoded proteins of the electron transport chain (ETC) [17]. HO-1 is generally found in the cytosol tethered to the ER, however, evidence indicates that HO-1 can localize to to mitochondria, including in a lung epithelial cancer cell line, although its role there remains unclear [18,19]. Additionally it has been suggested that there may be a metabolic link between the state of oxidative phosphorylation (oxphos) and heme [20]. To explore potentially unknown contributions of HO-1 to cell function, we used a lung epithelial cell line to investigate the effects of HO-1 loss on mitochondrial metabolism. We find that HO-1 positively contributes to oxidative phosphorylation via the ETC and TCA cycle.

## 2. Results

### 2.1. HO-1 Disruption Slows Growth and Reduces Oxidative Phosphorylation

**Figure 1:**
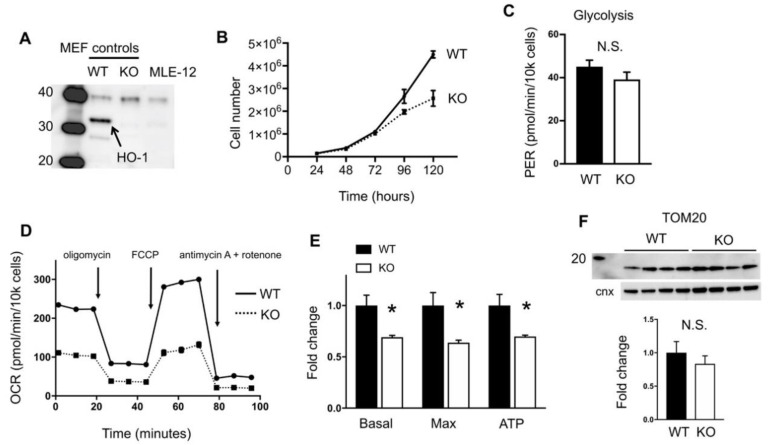
Heme oxygenase (HO-1) knockout cells have impaired growth and decreased mitochondrial respiration. (**A**), Western blot showing deletion of HO-1 in MLE-12 cells (far right). Positive (wild-type (WT)) and negative (knockout (KO)) controls from mouse embryonic fibroblasts (MEF) [23] were included. (**B**), Growth curves of WT and KO MLE-12 cells. (**C**), Glycolysis was measured in the Seahorse. PER, proton efflux rate. (**D**), Representative trace of the mitochondrial stress test measured in the Seahorse. OCR, oxygen consumption rate. Arrows indicate injection time of drugs. (**E**), Basal and maximal respiration, and ATP production were determined with the mitochondrial stress test. (**F**), Western blot of TOM20 (top) normalized to calnexin loading control (bottom). Molecular weight of 20 kDa is shown. * *p* < 0.05 vs. WT. Error bars are standard error of the mean (SEM).

In order to better define the cellular role of HO-1, we generated an HO-1 null MLE-12 cell line using CRISPR. This cell line has been used as in vitro models of lung alveolar type II cells to understand the negative effects of hyperoxic exposure in the lung, which is characterized by arrested alveolar growth [21,22]. As HO-1 is induced in the lung and in vitro in response to hyperoxia and other stresses, we wished to better understand the role of HO-1 in these cells. Previously we generated mouse embryonic fibroblast cells (MEFs) that were from wild-type (WT) or HO-1 global knockout (KO) mice [23], and we used these as controls for expression of HO-1. We confirmed by western blot that our MLE12 HO-1 knockout cell line is HO-1-deficient (Figure 1A). To determine the contribution of HO-1 to overall cellular fitness we examined growth rates of WT and KO MLE-12 cells and found that loss of HO-1 is associated with reduced growth (Figure 1B).

Our group and others have shown that hyperoxia-exposed MLE-12 cells have altered metabolism [21] thus we wished to know if HO-1 contributes to metabolic programs in these cells. Using a Seahorse Bioanalyzer, we conducted a glycolytic rate assay (Agilent) which measures acidification of the media due to proton release from glycolysis. We found no difference in glycolysis between WT and KO cells (Figure 1C). Next we interrogated oxidative phosphorylation with a mitochondrial stress test, measuring the oxygen consumption rate (OCR) over time in response to various drugs. We found that HO-1 knockout cells have reduced oxphos activity including loss of basal respiration, reduced maximal respiration in response to the uncoupler FCCP, and less ATP production (Figure 1D,E). Loss of mitochondria in KO cells was not likely, as levels of an outer mitochondrial resident protein, TOM20, remained unchanged (Figure 1F).

### 2.2. Loss of HO-1 Restricts Flow of Electrons in the Electron Transport Chain

**Figure 2:**
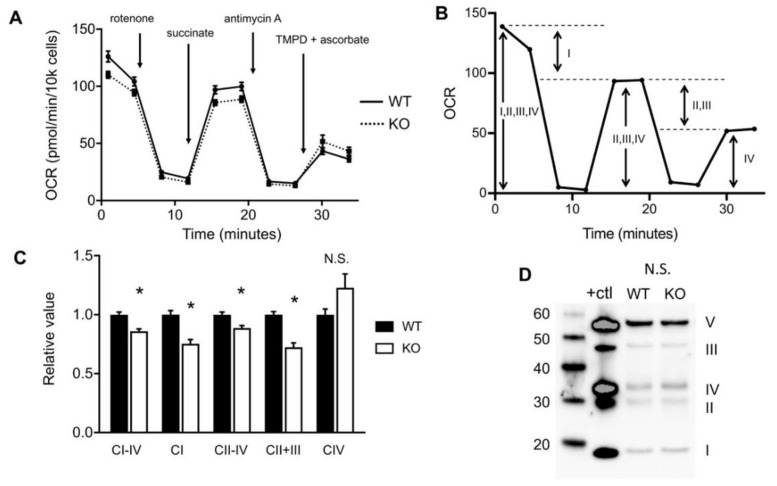
HO-1 knockout cells have reduced electron flow across the electron transport chain ETC. (**A**), Representative trace of the electron flow assay with injection strategy, measured with the Seahorse. (**B**), Illustration of parameters used to calculate the activities of each complex. (**C**), Relative activities of WT and KO ETC complexes. (**D**), Western blot of WT and KO cells using a five-antibody cocktail, detecting one protein from each ETC Complex. A positive control (+ctl) was provided by the manufacturer. Molecular weight ladder (20–60 kDa) is shown. OCR, oxygen consumption rate. * *p* < 0.05 vs. WT. Error bars are SEM.

The observed reduction in oxphos of HO-1 knockout cells prompted us to investigate which components of mitochondrial respiration may be targeted. First, we interrogated the electron transport chain since there are many heme and iron sulfur cofactors that are employed as electron carriers. Loss of heme catalysis by HO-1 or potential disruptions in heme homeostasis could be revealed by defective electron flow through the ETC. To address this, using a Seahorse Bioanalyzer, we conducted an electron flow experiment interrogating complexes together and individually. We used a series of drug injections designed to halt the flow of electrons at specific complexes and to supply substrates to regenerate activity [24]. The initial OCR reflects the activities of Complexes I through IV. Injection of rotenone halts the flow of electrons at Complex I, thus the difference in OCR before and after inhibition was used to determine total electron flow (Figure 2A,B). We found that flow of electrons across complexes I-IV was reduced in KO cells (Figure 2C). Injection of succinate restarts flow of electrons at Complex II and continues through Complex IV and we found this activity was also reduced in KO cells (Figure 2C). This enabled us to determine the contribution to electron flow of Complex I alone, which was reduced KO cells (Figure 2C). After inhibition of Complex III via injection of antimycin A, the addition of tetramethyl-*p*-phenylenediamine (TMPD) and ascorbate enables electron flow at Complex IV, which was unchanged in KO cells (Figure 2C). In short, the activities of Complex I and II are reduced in KO cells, however we are unable to resolve the activity of Complex III alone with this injection scheme. Apart from activities, it appears unlikely that HO-1 knockout cells are missing structural components of the ETC. Using an antibody cocktail that recognizes the most labile subunit of each of the five ETC complexes, we found no loss of these proteins in HO-1 knockout cells (Figure 2D).

### 2.3. HO-1 Knockout Cells Have Altered Mitochondrial Fuel Utilization

**Figure 3:**
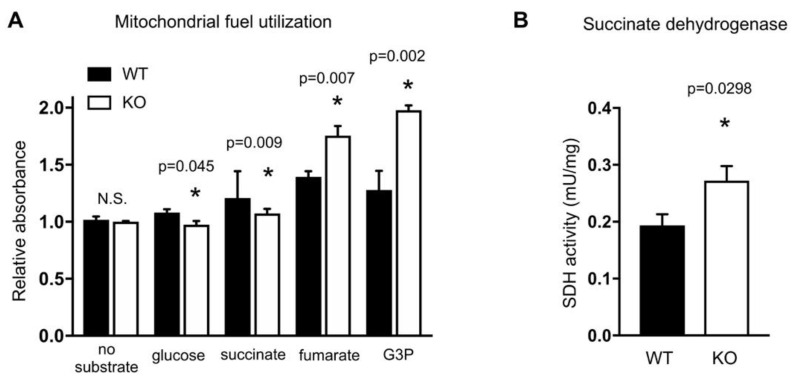
HO-1 knockout cells have altered mitochondrial fuel utilization. (**A**), Utilization of glycolytic and TCA cycle metabolites by WT and KO mitochondria was determined using the Biolog MitoPlate. (**B**), Succinate dehydrogenase activity was determined for WT and KO cells. * *p* < 0.05 vs. WT. Error bars are SEM.

We wished to know if the reduced mitochondrial respiration observed in HO-1 knockout cells extends beyond ETC dysfunction. Therefore, we used Biolog MitoPlate assays to assess the utilization of various substrates by mitochondria in WT and KO cells. The assay measures the rate of electron flow into and through the ETC from substrates that produce NADH and FADH_2_. Electrons enter either Complex I or II, and a dye that acts as a terminal electron acceptor changes color upon reduction. KO cells exhibit reduced glucose utilization (Figure 3A), which is consistent with observed reduced oxphos (Figure 1). Interestingly, we found that KO cells have reduced utilization of succinate (Figure 3A), which is consistent with our Seahorse electron flow data indicating reduced Complex II activity. Complex II is also known as succinate dehydrogenase (SDH) and participates in both the ETC and the TCA cycle. We also found that KO cells had increased utilization of glycerol 3 phosphate (G3P) compared to WT (Figure 3A). The G3P shuttle oxidizes NADH from glycolysis and contributes to the production of ATP using oxidative phosphorylation. G3P is oxidized at the inner mitochondrial membrane by mitochondrial glycerol 3 phosphate dehydrogenase (mGPDH) to generate dihydroxyacetone P (DHAP). Electrons are shuttled by mGPDH via FADH2 to reduce ubiquinone to ubiquinol, where they enter the ETC at Complex III and contribute to the proton gradient [25]. The increased utilization of G3P in KO cells may represent a mechanism to increase the proton gradient in compensation for reduced SDH activity. We did not detect changes in expression levels of mitochondrial or cytosolic GPDH mRNA by Taqman.

The first and rate-limiting step in heme biosynthesis is the condensation of the TCA cycle intermediate succinyl-CoA with glycine, catalyzed by aminolevulinic acid synthase (ALAS). Here we see in KO cells that utilization of fumarate was increased. Interestingly, an early study indicated that heme and fumarate metabolism are linked. The authors show that when heme synthesis was upregulated, it led to incorporation of radiolabeled fumarate into heme via succinate and succinyl-CoA [20]. More recently it has been shown that fumarate hydratase (FH) and HO-1 are synthetically lethal [26,27]. FH deficient cells accumulated fumarate and succinate and had increased expression of HO-1. When HO-1 expression was silenced in FH deficient cells there was significant loss of viability. The absence of HO-1 in our cells reaffirms this relationship, in which the balance is shifted back toward increased fumarate utilization by the TCA cycle.

Reduced succinate utilization and increased fumarate utilization in KO cells suggested a defect in SDH activity, which was indicated by the Seahorse assay (Figure 2), thus we conducted an in vitro assay of SDH activity (SDH kit, Abcam). In this assay, cells are lysed, succinate substrate is added in vitro, and oxidation to fumarate is measured by the transfer of electrons to an artificial electron acceptor and a colorimetric change. Surprisingly, HO-1 knockout cells exhibited increased SDH activity measured this way (Figure 3B). We are not sure how to reconcile these differences, but they may be explained by the nature of the different assays. In particular the Seahorse electron flow assay and the Biolog MitoPlate interrogate more holistic mitochondrial activity in the context of other complex activities whereas, in contrast, the Abcam assay is a purely in vitro assessment of succinate oxidation.

### 2.4. Iron Homeostasis in HO-1 Knockout MLE-12 Cells is Maintained

**Figure 4:**
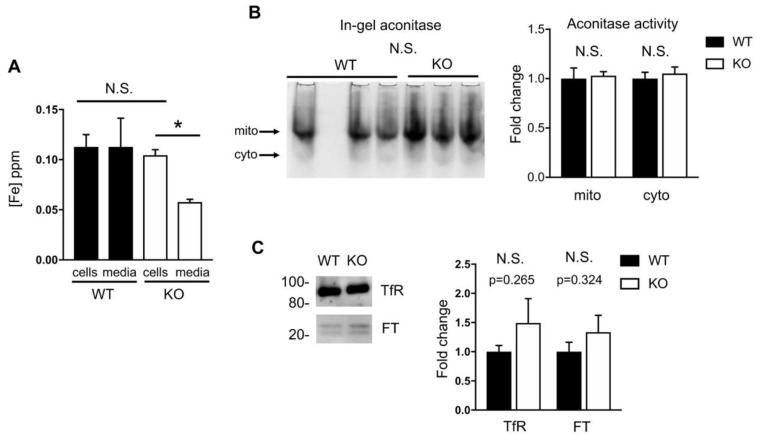
Iron homeostasis is preserved in HO-1 knockout cells. (**A**), Non-heme iron (ppm, parts per million) was measured using ICPOES for WT and KO. Intracellular iron was measured (cells) as was secreted iron (media). (**B**), In-gel assay measuring mitochondrial (mito) and cytosolic (cyto) aconitase activity in WT and KO cells. Quantification of band intensity shown at right. (**C**), Representative Western blot of transferrin receptor and ferritin in WT and KO cells (left), normalized to calnexin and quantified (right). * *p* < 0.05 vs. WT. Error bars are SEM.

Next, we wanted to know if MLE-12 cells lacking HO-1 have altered iron handling, since iron is released upon heme degradation. It has been suggested that one role of HO-1, apart from its antioxidant effects, is to support the intracellular supply of iron. Lack of heme breakdown results in accumulation of heme [18] and, we hypothesized, reduction in non-heme iron. However, when we measured non-heme iron, we saw no difference in cellular iron between WT and KO cells (Figure 4A). It has previously been shown that loss of HO-1 in fibroblasts resulted in increased iron uptake and decreased iron efflux [28]. We therefore examined non-heme iron that was secreted into the media and found that KO cells secreted less iron than WT cells.

We also examined biological properties that reflect iron availability in the cell and first conducted an in-gel aconitase enzymatic assay. Both cytosolic and mitochondrial aconitases are dependent on FeS clusters for their activity, converting citrate to isocitrate [29]. We see no loss of activity of cytosolic nor mitochondrial aconitase in KO cells compared to WT (Figure 4B), suggesting that iron is not limiting. Next we examined protein levels of transferrin receptor and ferritin as a proxy for iron levels [30]. Transferrin receptor is responsible for importing iron and its protein levels decrease when iron is plentiful. Ferritin is the major storage site for iron in the cell; when cells are replete with iron ferritin expression is high, and when iron is low ferritin expression is reduced. We observed no change in either of these proteins that reflect the level of intracellular iron. Taken together our data indicate that MLE12 cells lacking HO-1 do not have altered intracellular supply of iron.

## 3. Discussion

In cultured mouse lung epithelial cells, we show that disruption of HO-1 results in reduced oxphos via the ETC and the TCA cycle, through disruption of succinate dehydrogenase. We surmise that this is a consequence of excess heme, as it is not degraded in the absence of HO-1, and accumulation of succinate. It has been shown that inhibition of HO-1 increases mitochondrial heme content [18]. Indeed when succinate levels are high, membrane potential is high and electron flow is slow because ADP is limiting [31]. This is consistent with our observed reduced ATP production in KO cells (Figure 1E) and reduced electron flow (Figure 2). Kurumada found that when heme synthesis is upregulated, fumarate is incorporated, via succinate, into succinyl-CoA, reversing this portion of the TCA cycle [20]. Our results demonstrate the opposite scenario in which HO-1 loss leads to increased fumarate utilization, shifting the equilibrium in the forward direction. This part of the TCA cycle, or SDH specifically, may be particularly sensitive to levels of heme. Indeed the biosynthesis, trafficking, and degradation of heme are known to be heavily regulated [32]. Excess heme is known to inhibit the transcription, translation, and localization of ALAS to mitochondria [33,34,35], thus it is possible that HO-1 knockout cells accumulate succinyl-CoA and/or succinate but are less able to utilize them. The TCA cycle is the major source of mitochondrial NADH. When NADH is exhausted, as expected here with reduction of oxphos (Figure 1D), SDH activity is reduced [36]. This is consistent with our observed reduction in SDH activity with two assays using intact mitochondrial complexes. The increased SDH activity of KO cells observed with the Abcam kit may represent a simplified measure of SDH in the absence of other partners. This discrepancy may warrant further exploration using metabolomics to more precisely understand the alteration in the TCA cycle.

We also examined iron homeostasis in WT and KO cells by several independent methods. Most directly, we measured the amount of non-heme iron in cells and found no difference between WT and KO. Previously it was shown that HO-1 loss in fibroblasts resulted in increased iron uptake and decreased efflux [28]. In KO cells we did measure reduced iron secreted into the media but did not see increased iron in the cells. We do not fully understand this observation and it may be unique to specialized lung epithelial cells compared to fibroblasts. Ferris and colleagues also showed that mouse livers from HO-1 knockout mice had accumulated total iron; however, this includes heme iron, which is known to be increased [18,37,38]. Independently, we observed no changes in activity of the aconitases, nor in the protein levels of transferrin receptor and ferritin, all of which are regulated by iron. Taken together we suggest that, in lung epithelial cells, the contribution of HO-1 to cellular homeostasis may be more about regulation of heme than iron per se.

HO-1 has been shown to be protective in many pathologies including atherosclerosis, neurological disorders, metabolic syndrome, immunity and inflammation, and cancer [39]. Incidentally mitochondrial quality control is also linked to the above disease states. This highlights the need to understand the contributions of HO-1 to metabolism [40]. Here we show that HO-1 supports cell growth and mitochondrial function, by contributing to the activity of the ETC and the TCA cycle. HO-1 is evolutionarily conserved and acts to regulate the ancestral and ubiquitous heme cofactor. Thus the role of HO-1 may vary by cell and tissue type, partially explaining its pleiotropic contributions at the organismal level. Determining the precise metabolic role of HO-1 may provide insights into therapies and nutritional interventions to ameliorate diseases.

## 4. Materials and Methods

### 4.1. Cell Lines and Culture

MLE-12 cells from ATCC were maintained with 5% CO_2_, at 37 °C, in DMEM/F12 media containing 2% FBS, insulin (5 μg/mL), transferrin (10 μg/mL), sodium selenite (30 nM), hydrocortisone (10 nM), β-estradiol (10 nM), HEPES (10 nM), glutamine (2 mM) and 1% P/S (100 units/mL penicillin and 100 µg/mL streptomycin). To generate HO-1 knockout cells we took a two-step approach according to the manufacturer’s instructions (Sigma Aldrich, St. Louis, MO, USA). Using lentiviral transduction we first generated a cell line constitutively expressing Cas9, selecting blastocidin resistance (LVCAS9BST). We next transduced lentivirus targeting *hmox1* (MMPD0000017721; TATGTAAAGCGTCTCCACG), selected with puromycin and sorted into 96 well plates by FACS for clonal isolation. Throughout this work we designated the cell line expressing Cas9 as “WT” to allow a more direct comparison to KO cells also expressing Cas9. Growth curves were conducted by counting viable cells identified by trypan blue exclusion. All experiments were done in biological triplicate, at minimum.

### 4.2. Detection of Proteins

Proteins were isolated with RIPA buffer plus protease inhibitor cocktail (Sigma, P8340) and a tissue grinder for 5 s at 25,000 rpm (IKA; T25 digital Ultra Turrax, Staufen, Germany). Protein concentration was determined with the Pierce BCA assay kit (ThermoFisher 23225, Waltham, MA, USA). Proteins were separated using sodium dodecyl sulfate polyacrylamide gel electrophoresis (SDS-PAGE; ThermoFisher), transferred to PVDF membranes (Millipore, Burlington, MA, USA) and blotted with the following primary antibodies. HO-1 (in-house, need ref); TOM20 (Cell Signaling 42406, Danvers, MA, USA); total rodent oxphos antibody cocktail (Abcam ab110413, Cambridge, UK); calnexin (Enzo, ADI-SPA-860-F, Farmingdale, NY, USA); transferrin receptor (ThermoFisher 13-6800); ferritin (Abcam ab75973). Horseradish peroxidase conjugated secondary antibodies were used for visualization with Luminata Crescendo (EMD Millipore) or SuperSignal West Femto substrate (ThermoFisher) using the ChemiDoc Touch Imaging System (BioRAD, Hercules, CA, USA).

### 4.3. Mitochondrial Respiration and Glycolysis

Mitochondrial respiration and glycolytic function of WT and HO-1 knockout cells were measured in a XF^e^24 Seahorse Bioanalyzer and data were analyzed according to the manufacturer’s calculations (Agilent; Mitochondrial Stress Test and Glycolytic Rate Assay, Santa Clara, CA, USA). Cells were seeded the day prior to the assay. In order to account for biological batch to batch variability in cell growth overnight, immediately prior to the assay cells were confirmed to be evenly seeded and one well from each condition was counted by trypan blue exclusion and used for normalization. Final concentrations of injected reagents were: 1 μM oligomycin; 2 μM FCCP (carbonyl cyanide-4 (trifluoromethoxy) phenylhydrazone); 5 μM each rotenone and antimycin A; 10 mM glucose; and 50 mM 2-DG (2-deoxy-glucose).

### 4.4. Electron Flow

Cells were plated and normalized as above and the XF^e^24 Seahorse Bioanalyzer was used to measure OCR. Activity was assessed in 1x MAS buffer (described in XF Plasma Membrane Permeabilizer (PMP) Guide; Agilent) including 10 mM pyruvic acid, 2 mM malate, 4 μM FCCP, 4 mM ADP, 0.2% (*w*/*v*) fatty acid-free BSA and 1 nM PMP reagent [41]. Final concentrations of injected reagents were: 2 μM rotenone, 10 mM succinate, 1.5 μg/mL antimycin A, 10 mM ascorbate, and 100 μM tetramethyl-*p*-phenylenediamine (TMPD).

### 4.5. Biochemical Assays

Mitochondrial substrate utilization was conducted according to the manufacturer’s instructions (Biolog, Hayward, CA, USA) in which viable cell number was first determined by trypan blue exclusion, and plated in Biolog Mitoplates. A 96 well plate reader was used in kinetic mode at OD590 over 4 h and the relative amount of substrate utilization was determined by the change in absorbance over this time period. To measure total non-heme iron, cells at 70% confluency were utilized. Media was collected 1:1 with 3 N HCl/10% trichloroacetic acid (TCA); cells were washed with PBS, scraped into 1.0 mL 3 N HCl/10% TCA and stored at room temperature. For analysis, after hydrolysis at 70 °C for 24 h precipitating heme in 10% TCA, iron (non-heme) concentrations in the supernatant were determined using ICPOES (Model Optima 4300D, Perkin Elmer, Norwalk, CT, USA) operated at a wavelength of 238.204 nm. The in-gel aconitase activity assay was conducted as described [42], loading 70 μg protein per well. Succinate dehydrogenase activity was determined according to the manufacturer’s instructions (Abcam, ab228560).

### 4.6. Statistics

Statistical analyses were performed using GraphPad Prism 7. Results are expressed as mean ± SEM. Statistical significance was determined with a t-test between the means of two groups when *p* < 0.05.

## 5. Conclusions

In cultured mouse lung epithelial cells, we demonstrate that HO-1 contributes to cellular metabolism at the electron transport chain and the TCA cycle. This indicates a specific role for HO-1 in promoting metabolic homeostasis, in addition to its known antioxidant capacities.

## Abbreviations

ETC	Electron transport chain
HO-1	Heme oxygenase 1
OCR	Oxygen consumption rate
Oxphos	Oxidative phosphorylation
ROS	Reactive oxygen species
SDH	Succinate dehydrogenase
